# Self-Supervised Adversarial Learning with a Limited Dataset for Electronic Cleansing in Computed Tomographic Colonography: A Preliminary Feasibility Study

**DOI:** 10.3390/cancers14174125

**Published:** 2022-08-26

**Authors:** Rie Tachibana, Janne J. Näppi, Toru Hironaka, Hiroyuki Yoshida

**Affiliations:** 1 3D Imaging Research, Department of Radiology, Massachusetts General Hospital and Harvard Medical School, 25 New Chardon Street, Suite 400C, Boston, MA 02114, USA; 2 Information Science & Technology Department, National Institute of Technology, Oshima College, 1091-1 Komatsu Suo-Oshima, Oshima, Yamaguchi 742-2193, Japan

**Keywords:** self-supervised learning, generative adversarial network, electronic cleansing, virtual colonoscopy, artificial intelligence

## Abstract

**Simple Summary:**

Electronic cleansing (EC) is used for performing a virtual cleansing of the colon on CT colonography (CTC) images for colorectal cancer screening. However, current EC methods have limited accuracy, and traditional deep learning is of limited use in CTC. We evaluated the feasibility of using self-supervised adversarial learning to perform EC on a limited dataset with subvoxel accuracy. A 3D generative adversarial network was pre-trained to perform EC on the CTC datasets of an anthropomorphic colon phantom, and it was fine-tuned to each input case by use of a self-supervised learning scheme. The visually perceived quality of the virtual cleansing by this method compared favorably to that of commercial EC software on the virtual 3D fly-through examinations of 18 clinical CTC cases. Our results indicate that the proposed self-supervised scheme is a potentially effective approach for addressing the remaining technical problems of EC in CTC for colorectal cancer screening.

**Abstract:**

Existing electronic cleansing (EC) methods for computed tomographic colonography (CTC) are generally based on image segmentation, which limits their accuracy to that of the underlying voxels. Because of the limitations of the available CTC datasets for training, traditional deep learning is of limited use in EC. The purpose of this study was to evaluate the technical feasibility of using a novel self-supervised adversarial learning scheme to perform EC with a limited training dataset with subvoxel accuracy. A three-dimensional (3D) generative adversarial network (3D GAN) was pre-trained to perform EC on CTC datasets of an anthropomorphic phantom. The 3D GAN was then fine-tuned to each input case by use of the self-supervised scheme. The architecture of the 3D GAN was optimized by use of a phantom study. The visually perceived quality of the virtual cleansing by the resulting 3D GAN compared favorably to that of commercial EC software on the virtual 3D fly-through examinations of 18 clinical CTC cases. Thus, the proposed self-supervised 3D GAN, which can be trained to perform EC on a small dataset without image annotations with subvoxel accuracy, is a potentially effective approach for addressing the remaining technical problems of EC in CTC.

## 1. Introduction

Colorectal cancer is the second-leading cause of cancer-related deaths in the United States [1]. However, it is known that early detection and removal of benign precursor lesions prevent the development of colorectal cancer. Computed tomographic colonography (CTC) provides a safe and accurate method for examining the complete region of the colon for those precursor lesions and early cancers. Indeed, CTC is recommended by the American Cancer Society and the U.S. Preventive Services Task Force as an option for colon cancer screening [2,3].

CTC uses orally administered contrast agents to indicate retained fluid and feces that could otherwise obscure or imitate polyps and cancers on the CTC images [4]. To visualize the complete region of the colon without the residual materials, an image processing method called electronic cleansing (EC) is used for computationally removing the contrast-enhanced (tagged) fecal materials from the CTC images, thus performing a “virtual cleansing” of the colon [5]. This enables a virtual three-dimensional (3D) fly-through reading of the colon, similar in appearance to that of a conventional optical colonoscopy examination.

Early EC methods consisted of mathematical models based on an explicit segmentation of the fecal materials on CTC images [6,7,8]. Such methods produce many image artifacts [5] because the virtual cleansing is performed by subtracting the segmented regions from the images. Therefore, the accuracy of EC is limited to the underlying physical voxel resolution of the CT image volume, whereas realistic EC outcomes require resolving the output CT values with subvoxel accuracy. Subsequent methods attempted to address this issue by sophisticated mathematical modeling of the multiple material fractions contained within each voxel [9,10,11,12,13,14]. However, these methods were still vulnerable to uncertainties due to the ambiguity of CT values and due to pseudo-enhancement effects of the contrast agent on the CTC images [15]. Although the image artifacts generated by EC can be subdued by post-processing of the EC images [6], ultimately, clinicians have considered the potential benefit of these segmentation-based EC methods to be outweighed by the image artifacts that they generate [16,17,18].

Therefore, instead of employing explicit mathematical modeling, the most recent approaches have been based on machine learning of the virtual cleansing. Previously, we developed a multi-material scheme, where machine learning was used to classify each voxel into the specific material or unique partial-volume mixture of materials contained within that voxel [19]. The multi-material analysis was performed based on local radiomic features by use of a random forest classifier [20], or directly on the local CT image values by use of deep convolutional neural networks [19]. However, these machine learning methods were still based on image segmentation and traditional supervised learning, which limited their accuracy to that of the underlying physical voxel resolution. They also exhibit the drawback that the input clinical CTC cases do not have the corresponding desired EC output image volumes that are required for the training of machine learning models based on supervised learning.

In this study, we explored the technical feasibility of developing a novel self-supervised 3D generative adversarial network (3D GAN) EC scheme to address these problems. The proposed scheme has two specific advantages over the previous EC methods. First, the use of a 3D GAN provides a mechanism for translating uncleansed CTC image volumes directly into their corresponding virtually cleansed image volumes, without undertaking an explicit segmentation process, and thus, the accuracy of EC is not limited to the underlying voxel resolution. Second, the use of a GAN with self-supervised learning enables effective training with a smaller number of annotated CTC training cases than what is required by traditional supervised machine learning models [21,22,23].

To demonstrate the feasibility of the proposed self-supervised adversarial learning approach, we performed a pilot evaluation of the performance of our 3D-GAN EC scheme by use of a limited dataset based on an anthropomorphic phantom and 18 clinical CTC cases. The quality of the virtual cleansing (hereafter called *cleansing quality*) by the 3D-GAN EC scheme was compared with that of commercial EC software.

## 2. Materials and Methods

### 2.1. CTC Datasets

This retrospective study was reviewed and approved by the Mass General Brigham Institutional Review Board. To simulate the appearances of contrast-enhanced (fecal-tagging) bowel contents in combination with known ground truth, an anthropomorphic colon phantom (Phantom Laboratory, Salem, NY, USA) that had been designed to imitate a human colon in abdominal CT scans was partially filled with 300 mL of a mixture of saline, non-ionic iodinated contrast agent (OMNIPAQUE^TM^ (iohexol) Injection 300 mgI/mL, GE Healthcare, Chicago, IL, USA), aqueous fiber (30 g of psyllium), and ground foodstuff (10 g of cereal). The contrast agent was applied in three separate sessions in low (20 mg/mL), moderate (40 mg/mL), and high (60 mg/mL) concentrations to simulate the different appearances of tagged fecal materials in clinical CTC cases. The native (empty) and partially filled versions of the colon phantom were scanned by use of a dual-energy CT (DE-CT) scanner (SOMATOM Definition Flash, Siemens Healthcare, Erlangen, Germany) in single-energy mode with 120 kVp tube voltage, 0.6-mm slice thickness, 0.61 mm pixel spacing, and 0.6-mm reconstruction interval. After the CT scans, the phantom CTC image volumes were registered to match spatially at each voxel, based on manually-determined offsets between the datasets. Hereafter, we will refer to these CT image volumes as phantom CTC datasets.

In addition, for a clinical evaluation, 18 patients were prepared for a CTC examination with a non-cathartic preparation. The bowel preparation regimen consisted of oral ingestion of 50 mL of iodinated contrast agent (Gastrografin, Bracco Diagnostics, Princeton, NJ, USA) on the day before and again two hours before the CT scan. The patients were scanned in two (supine, prone, and/or decubitus) positions by use of a DE-CT scanner (SOMATOM Definition Flash) in dual-energy mode with 140 kVp and 80 kVp tube voltages, 1.0-mm slice thickness, 0.57–0.76 mm pixel spacings, and 0.7-mm reconstruction interval. To obtain single representative CT image volumes corresponding to the parameters of the phantom CTC dataset, the clinical CT image volumes of the patients were reconstructed from the DE-CT scans as mixed-energy image volumes corresponding to the 120 kVp tube voltage. Hereafter, we will refer to these CT image volumes as clinical CTC cases.

The CT image volumes of the phantom CTC datasets and the clinical CTC cases were interpolated in the axial direction to an isotropic voxel resolution, according to their pixel spacing.

### 2.2. Extraction of Volumes of Interest (VOIs)

To obtain paired VOIs for the training and evaluation of the 3D GAN in the following sections, we extracted a total of 400 VOIs with 128 × 128 × 128 voxels from matching lumen centerline locations of the registered image volumes of the CTC phantom datasets, acquired without (native phantom, 100 VOIs) and with the low (100 VOIs), moderate (100 VOIs), and high (100 VOIs) concentrations of the contrast agent.

For each clinical CTC case, we also extracted 100 VOIs along the lumen centerlines of the CTC image volumes of each case. The calculations of the lumen centerlines and the extractions of VOIs were performed automatically by use of our previously developed computer-aided detection software [24]. These VOIs were used for the self-supervised adversarial training of the 3D GAN, as described in Section 2.4.

### 2.3. 3D GAN for EC

**Figure 1:**
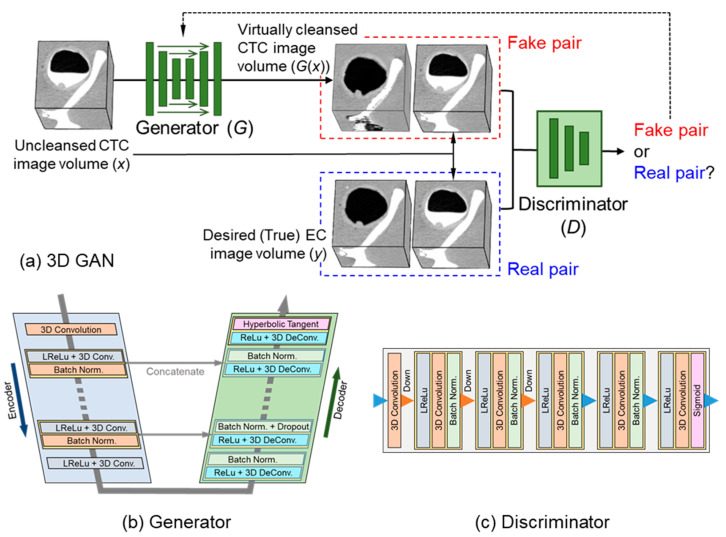
(**a**) Overview of the training process in the 3D GAN. (**b**) Architecture of the generator in the 3D GAN. (**c**) Architecture of the discriminator in the 3D GAN.

Figure 1a shows the architecture and the training process of the 3D GAN that we used in this study. The architecture of the 3D GAN is based on the design of the 2D pix2pix GAN [25] that was originally developed for manipulating 2D photos.

Our 3D GAN is composed of a generator network (*G*) and a discriminator network (*D*) that make use of 3D-convolution kernels. The overall architecture of *G* (Figure 1b and Table A1) is based on the encoder-decoder architecture of U-Net [26]. It is composed of N successive 3D-convolutional downsampling and N corresponding successive 3D-deconvolutional upsampling layers. The purpose of *G* is to convert the original uncleansed CTC image volume (*x*) into the corresponding virtually cleansed CTC image volume (*G*(*x*)).

The overall architecture of *D* (Figure 1c and Table A2) is based on the PatchGAN model of the 2D pix2pix GAN [25]. It has three two-stride 3D-convolutional layers, two 3D-convolutional layers, and a sigmoid function layer. The purpose of *D* is to differentiate a “fake pair” of the above *x* and *G*(*x*) from a “real pair” of x and the desired (true) EC image volume *y* to provide feedback to *G* during training.

The loss function of the 3D GAN can be expressed as

(1) $$\begin{array}{c} G^{*}=\arg\underset{G}{\min}\underset{D}{\;\max}[L_{\mathrm{GAN}}\left(G,D\right)+\lambda L_{1}\left(G\right)], \end{array}$$

where $L_{\mathrm{GAN}}$ is the standard conditional-adversarial loss function [27]

(2) $$L_{\mathrm{GAN}}\left(G,D\right)=E_{x,y}[\log D\left(x,y\right)]+E_{x}\left[\log\left(1-D\left(x,G\left(x\right)\right)\right)\right],$$

and $L_{1}$ is the loss function that encourages *G* to fool *D* by generating a virtually cleansed CTC image volume that is similar to the desired EC image volume, i.e.,

(3) $$L_{1}\left(G\right)=E_{x,y}\left[\|y-G{\left(x)\right\|}_{1}\right].$$

The trade-off between $L_{\mathrm{GAN}}$ and $L_{1}$ is controlled by the parameter λ of Equation (1).

### 2.4. Self-Supervised Learning of 3D GAN

The 3D GAN was pre-trained by use of traditional supervised learning based on the VOIs that were extracted, as described in Section 2.2, from the registered CTC datasets of the anthropomorphic phantom imaged without (native phantom) and with the contrast-enhanced simulated fecal materials (fecal-tagging phantom), where the VOIs of the native phantom were used for representing the desired EC image volumes for the supervised training. These VOI pairs are called *fixed-truth VOI pairs*.

**Figure 2:**
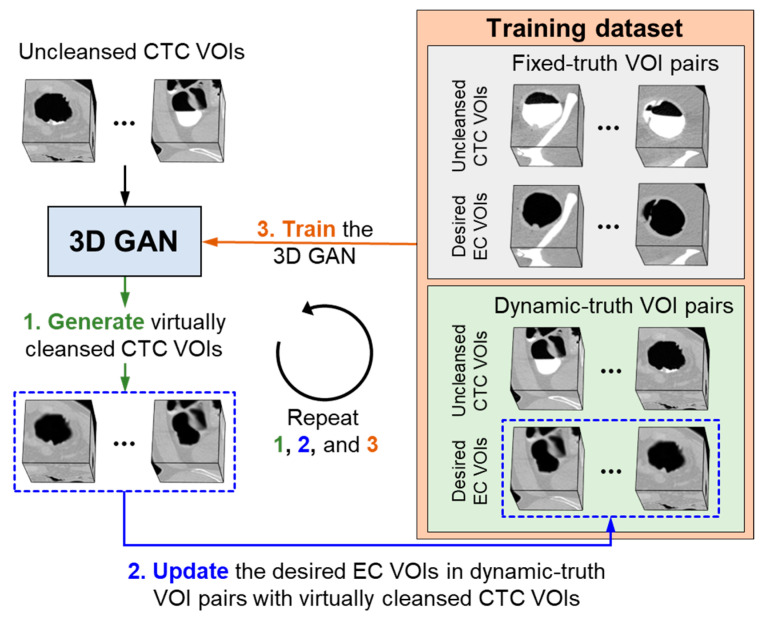
Overview of our proposed self-supervised learning for the training of the 3D-GAN EC scheme.

Figure 2 shows an overview of our proposed self-supervised learning scheme. The pre-trained 3D GAN is now fine-tuned by use of the self-supervised learning based on uncleansed CTC VOIs (illustrated at the top left corner of Figure 2), such as those that we extract from each clinical CTC case, as described in Section 2.1. The training dataset, which in the pre-training step, included only fixed-truth VOI pairs, is now expanded to include the uncleansed CTC VOIs as well. Because clinical CTC cases do not have the corresponding desired EC image volumes needed for the training of the 3D GAN, the initial desired EC VOIs corresponding to the uncleansed CTC VOIs are estimated by use of our previously developed fast random-forest EC scheme [28]. The uncleansed CTC VOIs are paired with these desired EC VOIs, and the resulting VOI pairs are called *dynamic-truth VOI pairs*. The dynamic-truth VOI pairs are illustrated at the bottom right corner of Figure 2.

The dynamic-truth VOI pairs are included in the training dataset for self-supervised learning together with the fixed-truth VOI pairs (illustrated at the top right corner of Figure 2) that were used in the pre-training step. The training of the 3D GAN is continued iteratively in a feedback loop, where both the fixed-truth and dynamic-truth VOI pairs, shown as the training dataset on the right half of Figure 2, are used for the training. Here, however, after each training iteration (indicated by step 3 in Figure 2), the desired EC VOIs of the dynamic-truth VOI pairs are updated by the application of the evolving 3D GAN itself to the uncleansed CTC VOIs (indicated by steps 1 and 2 in Figure 2).

Finally, after the training of the 3D GAN with multiple iterations, the generator of the 3D GAN is applied to the original clinical CTC case, which determines the final output of the 3D-GAN EC scheme for the case.

### 2.5. Implementation of the 3D-GAN EC Scheme

The 3D GAN was pre-trained with 200 fixed-truth VOI pairs extracted from the phantom CTC datasets. The fixed-truth VOI pairs were constructed by pairing the 100 VOIs extracted from the CTC dataset of the native phantom with the corresponding 100 uncleansed VOIs extracted from the CTC datasets of the fecal-tagging phantom acquired with low concentration and with the corresponding 100 uncleansed VOIs extracted from the CTC datasets of the fecal-tagging phantom acquired with a high concentration of the contrast agent (Section 2.2).

For the self-supervised training step, an additional set of 100 uncleansed VOIs was extracted from the input CTC case to construct the dynamic-truth VOI pairs. Thus, during the self-supervised training step, the 3D GAN was trained with a total of 300 fixed-truth and dynamic-truth VOI pairs.

For a review of the VOIs by the convolution networks, the original 16-bit CT values of the VOIs were clipped to a Hounsfield unit value range of [−1024, 1024], which was scaled to a value range of [−1, 1]. At each training iteration, the generator and discriminator networks of the 3D GAN were trained for 200 epochs using the Adam optimizer with a batch size of 3 and a learning rate of 0.00002, where the parameters of the networks were set to the same default values as those of the pix2pix GAN [25]. The experiments were carried out on a GeForce GTX 1080 Ti GPU (NVIDIA, Santa Clara, CA, USA).

### 2.6. Evaluation Methods

#### 2.6.1. Phantom Study: Objective Evaluation and Optimization of the 3D-GAN EC Scheme

For an objective evaluation and optimization of the self-supervised 3D-GAN EC scheme, the 3D GAN was first pre-trained with the fixed-truth VOI pairs derived from the phantom datasets acquired with low and high concentrations of the contrast agent, as described in Section 2.5. Then, 100 dynamic-truth VOI pairs were constructed by applying the self-supervised learning scheme to the uncleansed VOIs extracted from the CTC dataset of the fecal-tagging colon phantom imaged with a moderate concentration of the contrast agent (Section 2.2) that had not been used for the pre-training of the 3D GAN.

The cleansing quality in these 100 VOIs was quantified by use of the peak signal-to-noise ratio (PSNR) metric

(4) $$\mathrm{PSNR}=10\log_{10}\frac{{\mathrm{MAX}}^{2}}{\mathrm{MSE}},$$

where MAX=1024 Hounsfield units and MSE is the mean square error

(5) $$\mathrm{MSE}=\frac{1}{S^{3}}\overset{S}{\underset{i=0}{\sum }}\overset{S}{\underset{j=0}{\sum }}\overset{S}{\underset{k=0}{\sum }}{\left\{I\left(i,j,k\right)-J\left(i,j,k\right)\right\}}^{2}\;,$$

where *S* × *S* × *S* is the size of the VOI, I is the desired EC VOI (i.e., *y*), and J is the output VOI that is generated by *G* (i.e., *G*(*x*)). It should be noted that the PSNR values were calculated after the value rage of the VOIs was scaled back from [−1, 1] to [−1024, 1024].

To optimize the EC scheme, we estimated the cleansing quality for several numbers of the convolutional layers of *G* and over several training iterations of the self-supervised learning scheme. The statistical significance of the differences among the PSNR values was tested by use of the paired *t*-test.

#### 2.6.2. Clinical Study: Evaluation of the Cleansing Quality in Clinical CTC Cases

For evaluation of the cleansing quality in the clinical CTC cases by use of the self-supervised 3D-GAN EC scheme, the 3D GAN was first pre-trained with the fixed-truth VOI pairs from the phantom datasets, as described in Section 2.5. Then, for each of the 18 clinical CTC cases (Section 2.2), the dynamic-truth VOI pairs were constructed by applying the self-supervised learning scheme to the 100 VOIs extracted from the CTC datasets of the patient.

The cleansing quality of the 3D-GAN EC for these 18 clinical CTC cases was assessed on a CTC reading workstation by calculation of the mean number of the image artifacts observed on the virtual 3D fly-through examinations of the CTC datasets of the patients. The numbers of the image artifacts were compared with those generated by commercial EC software (AZE Virtual Place Fujin Raijin 310, Canon Medical Systems, Tochigi, Japan).

## 3. Results

### 3.1. Phantom Study

**Figure 3:**
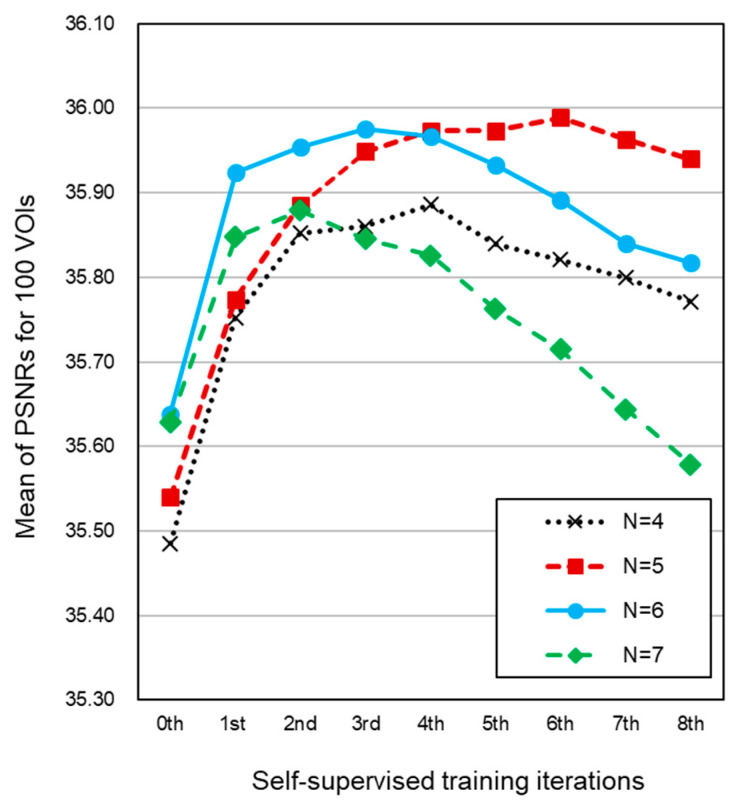
The mean value of the PSNR over 100 VOIs that were extracted from the fecal-tagging anthropomorphic colon phantom that was cleansed virtually by use of the proposed self-supervised 3D-GAN EC scheme. The values are shown for the initial supervised learning step and for the subsequent self-supervised training iterations for different numbers of convolutional and deconvolutional layers (N) of *G*. A high PSNR value indicates a higher cleansing quality than a low value.

Figure 3 shows the plots of the mean value of PSNR over the 100 dynamic-truth VOI pairs from the phantom after pre-training (0th iteration) and after the subsequent eight self-supervised training iterations. The value of N is the number of convolutional and deconvolutional layers used in the generator *G*. The graph shows that the best result was obtained by use of five layers (N = 5) with six self-supervised training iterations. In this case, the supervised training step yielded a mean PSNR of 35.54 ± 1.12, and after six self-supervised training iterations, the mean PSNR had increased to 35.99 ± 1.11.

Table 1 shows the *p*-values of the paired *t*-test on the PSNRs for the different numbers of layers and self-supervised training iterations in comparison to the optimized self-supervised 3D-GAN EC scheme (N = 5, six training iterations). The PSNR values obtained after the self-supervised training iterations were statistically significantly higher (*p* < 0.00001) than the PSNR values obtained from the pre-training (0th iteration), except for N = 7 at the sixth, seventh, and eighth training iterations.

### 3.2. Clinical Study

**Figure 4:**
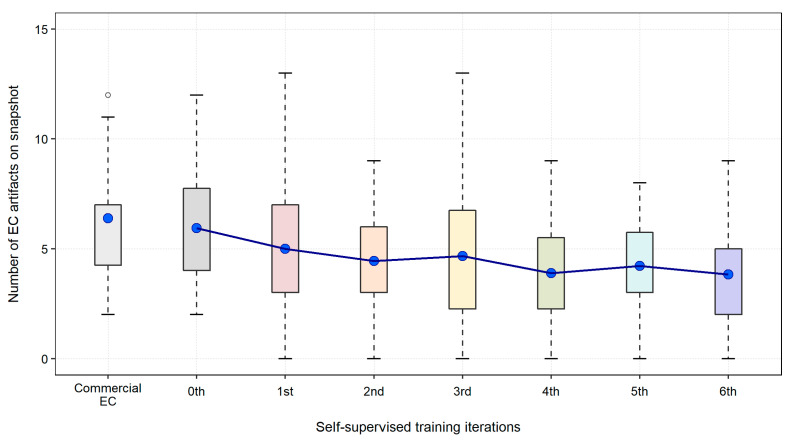
Comparison of the mean numbers of EC artifacts (indicated by blue circles) observed on the virtual 3D fly-through views of the virtually cleansed clinical CTC cases generated by the commercial EC software and over the successive iterations of the self-supervised training of our proposed 3D-GAN EC scheme (N = 5).

The boxplots in Figure 4 show the mean and standard deviation of the numbers of EC artifacts that were observed visually on the virtual 3D fly-through examinations of the 18 clinical CTC cases. The result is shown for the commercial EC software and over the different self-supervised training iterations of the 3D-GAN EC scheme at N = 5. The boxplot demonstrates how the artifacts are reduced gradually as the number of training iterations in the self-supervised learning increases.

**Figure 5:**
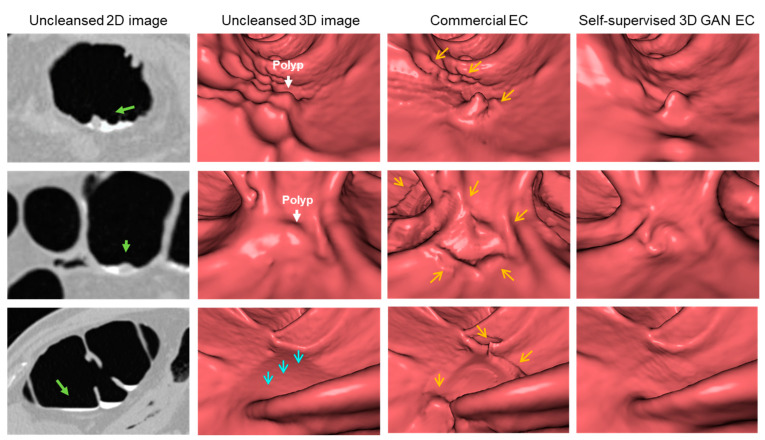
Visual comparison of the virtual cleansing by the self-supervised 3D-GAN EC and commercial EC. In the first column, the green arrows show the direction of the virtual camera in the virtual 3D fly-through views to the right. In the second column, the white arrows on the top and middle rows show locations of polyps partially submerged in residual feces, and on the bottom row, the cyan arrows indicate the location of a thin layer of fluid to be cleansed. In the third column, the orange arrows indicate locations of observed EC image artifacts by the commercial EC, which are not present on the EC images of the self-supervised 3D-GAN EC in the fourth column.

Finally, Figure 5 demonstrates the differences between the virtual 3D fly-through views generated by use of the self-supervised 3D-GAN EC scheme and by the commercial EC software. The top and middle rows show examples of polyps partially submerged in residual feces, and the bottom row shows an example of a thin layer of fluid. These examples are technically challenging to EC because they require the EC method to resolve between the three-material partial-volume boundaries of lumen air, soft tissue, and fecal tagging, while preserving the adjacent thin haustral folds. The images show that both EC methods are able to reveal the colon surface under the fecal tagging, but that the surface generated by use of the self-supervised 3D-GAN EC has fewer image artifacts than that generated by the commercial EC software. Additionally, the size and shape of the polyps are preserved more accurately by the use of the self-supervised 3D-GAN EC than by the commercial EC software.

## 4. Discussion

Early EC methods for CTC were based on mathematical segmentation-based models, which tend to generate artifacts on the resulting images. The most recent approaches have used machine learning to address the problems of the early EC methods. However, existing machine-learning-based EC methods are still based on a segmentation of the CTC images, and the available clinical CTC cases for the training of such methods are relatively small in number and do not have the desired EC output image volumes that are required for the traditional supervised training of standard machine learning models.

In this study, we explored the feasibility of using a novel self-supervised 3D GAN to address these problems. The use of a 3D GAN avoids the problems of image segmentation because the input CTC volume is transformed directly into the corresponding EC image volume with subvoxel accuracy. Moreover, the use of self-supervised learning with a GAN enables the training of the model with a much smaller number of cases than what is required by traditional supervised machine learning models. Despite the limited training dataset, our empirical results indicate that the resulting cleansing quality compares favorably with that of existing commercial EC software. It should be noted that the pre-training with an anthropomorphic phantom and the use of self-supervised learning also avoid the laborious annotation efforts that are required for the training of supervised machine-learning EC schemes.

The clinical CTC cases of this study were acquired initially by use of DE-CT scans and converted into a single mixed-energy CT volume to simulate conventional single-energy CTC. We only considered single-energy CTC to enable a fair comparison with the reference method of commercial EC software, which does not use dual-energy information. However, we have previously shown that incorporating dual-energy information into a machine-learning EC model improves the cleansing quality [19].

In the phantom study, the differences that were observed in the PSNR values between the different methods and training iterations are seemingly small. This is because less than 1% of the voxels of most VOIs are associated with fecal tagging and because most EC artifacts that are seen in 3D fly-through examinations are caused by an even smaller subset of tagged voxels that typically occur at the partial-volume boundaries of fecal tagging and other materials [19].

This technical feasibility study had several limitations. First, the number of test cases was not very large. However, it was large enough to demonstrate the feasibility and potential benefits of the self-supervised learning scheme. Second, the initial EC estimates of the self-supervised learning were obtained by use of an external EC method. Future studies should consider making the self-supervised learning scheme entirely self-contained. Third, the comparative evaluation with existing EC methods was limited to commercial EC software. Future studies should include testing with a larger number of EC methods to establish the relative performance of the method. Addressing these limitations in terms of a large-scale clinical follow-up study would strengthen our findings regarding the observed benefits of the proposed self-supervised learning scheme in EC for CTC.

## 5. Conclusions

We developed and evaluated the technical feasibility of a novel self-supervised 3D-GAN scheme to perform EC with subvoxel accuracy with a limited dataset in CTC. We showed that the scheme could be used to generate EC images that compare favorably in cleansing quality to those of existing commercial EC. The use of self-supervised learning also avoids the laborious annotation efforts required by current supervised machine learning solutions. Therefore, the proposed scheme is a potentially effective approach for addressing the remaining technical challenges of EC in CTC.

## Figures and Tables

**Table 1:** Paired *t*-test of the differences of the PSNRs for different numbers of convolutional layers, N, in comparison to the five convolutional layers of the optimized self-supervised 3D-GAN EC scheme.

	**0th**		**1st**		**2nd**		**3rd**		**4th**	
	**t**	***p*** **-Value**	**t**	***p*** **-Value**	**t**	***p*** **-Value**	**t**	***p*** **-Value**	**t**	***p*** **-Value**
N = 4	1.929	0.057	0.778	0.439	1.250	0.214	3.477	0.001	3.414	0.001
N = 6	−4.621	0.000	−6.610	0.000	−3.187	0.002	−1.294	0.199	0.279	0.781
N = 7	−4.121	0.000	−3.331	0.001	0.244	0.808	4.253	<0.0001	5.984	<0.0001
			**5th**		**6th**		**7th**		**8th**	
			**t**	***p*** **-Value**	**t**	***p*** **-Value**	**t**	***p*** **-Value**	**t**	***p*** **-Value**
N = 4			5.254	<0.0001	6.648	<0.0001	6.253	<0.0001	6.092	<0.0001
N = 6			1.808	0.074	4.255	<0.0001	5.107	<0.0001	4.987	<0.0001
N = 7			8.340	<0.0001	10.010	<0.0001	11.167	<0.0001	12.740	<0.0001

## Data Availability

Restrictions apply to the availability of these data. The private image datasets are not available online. The data generated by this study are available on request from the corresponding author.

## Appendix A

**Table A1:** Detailed architecture of the generator network of the 3D GAN in Figure 1b.

Layers	Kernel	Stride	Padding	Output Shape	Activation	Batch Norm.	Dropout
Input: Image				128 × 128 × 128 × 1			
Conv. Layer 1	4		2	1	64 × 64 × 64 × 64	LeakyReLU	
Conv. Layer 2	4	2	1	32 × 32 × 32 × 128	LeakyReLU	True	
Conv. Layer 3	4	2	1	16 × 16 × 16 × 256	LeakyReLU	True	
Conv. Layer 4	4	2	1	8 × 8 × 8 × 512	LeakyReLU	True	
Conv. Layer 5	4	2	1	4 × 4 × 4 × 512	LeakyReLU	True	
Conv. Layer 6	4	2	1	2 × 2 × 2 × 512	LeakyReLU	True	
Conv. Layer 7	4		2	1	1 × 1 × 1 × 512	ReLU	
Deconv. Layer 8	4	2	1	2 × 2 × 2 × 512		True	
Concatenate (Layer 8, Layer 6)							
Deconv. Layer 9	4	2	1	4 × 4 × 4 × 512	ReLU	True	True
Concatenate (Layer 9, Layer 5)							
Deconv. Layer 10	4	2	1	8 × 8 × 8 × 512	ReLU	True	True
Concatenate (Layer 10, Layer 4)							
Deconv. Layer 11	4	2	1	16 × 16 × 16 × 256	ReLU	True	
Concatenate (Layer 11, Layer 3)							
Deconv. Layer 12	4	2	1	32 × 32 × 32 × 128	ReLU	True	
Concatenate (Layer 12, Layer 2)							
Deconv. Layer 13	4	2	1	64 × 64 × 64 × 64	ReLU	True	
Concatenate (Layer 13, Layer 1)							
Deconv. Layer 14	4	2	1	128 × 128 × 128 × 1	Tanh		

**Table A2:** Detailed architecture of the discriminator network of the 3D GAN in Figure 1c.

Layers	Kernel	Stride	Padding	Output Shape	Activation	Batch Norm.
Input 1: Real Image				128 × 128 × 128 × 1		
Input 2: Fake Image				128 × 128 × 128 × 1		
Concatenate (Input 1, Input 2)						
Conv. Layer 1	4	2	1	64 × 64 × 64 × 64	LeakyReLU	
Conv. Layer 2	4	2	1	32 × 32 × 32 × 128	LeakyReLU	True
Conv. Layer 3	4	2	1	16 × 16 × 16 × 256	LeakyReLU	True
Conv. Layer 4	4	2	1	8 × 8 × 8 × 512	LeakyReLU	True
Conv. Layer 5	4	2	1	4 × 4 × 4 × 1	Sigmoid	
